# Data-driven subphenotyping uncovers ulcerative colitis subtype with high risk for relapse in Japan: a prospective multicenter cohort study

**DOI:** 10.1016/j.lanwpc.2026.101932

**Published:** 2026-07-24

**Authors:** Yohei Mikami, Hajime Yamazaki, Tadakazu Hisamatsu, Masakazu Nagahori, Taku Kobayashi, Maria Yonezawa, Shinichiro Shinzaki, Toshimitsu Fujii, Masayuki Saruta, Satoshi Motoya, Takayuki Yamamoto, Minoru Matsuura, Teppei Omori, Shunichi Fukuhara, Katsuyoshi Matsuoka

**Affiliations:** a Division of Gastroenterology and Hepatology, Department of Internal Medicine, Keio University School of Medicine, Tokyo, Japan; b Section of Clinical Epidemiology, Department of Community Medicine, Graduate School of Medicine, Kyoto University, Kyoto, Japan; c Department of Gastroenterology and Hepatology, Kyorin University School of Medicine, Tokyo, Japan; d Department of Gastroenterology, Health Science Research and Development Center, Institute of Science Tokyo, Tokyo, Japan; e Center for Advanced IBD Research and Treatment, Kitasato University Kitasato Institute Hospital, Tokyo, Japan; f Institute of Gastroenterology, Tokyo Women's Medical University, Tokyo, Japan; g Department of Gastroenterology, Hyogo Medical University School of Medicine, Nishinomiya, Hyogo, Japan; h Department of Gastroenterology and Hepatology, Institute of Science Tokyo, Tokyo, Japan; i Division of Gastroenterology and Hepatology, Department of Internal Medicine, The Jikei University School of Medicine, Tokyo, Japan; j Inflammatory Bowel Disease Center, Sapporo-Kosei General Hospital, Sapporo, Japan; k Inflammatory Bowel Disease Center and Department of Surgery, Yokkaichi Hazu Medical Center, Yokkaichi, Mie, Japan; l Department of Gastroenterology and Hepatology, Kyorin University School of Medicine Suginami Hospital, Tokyo, Japan; m Department of Health Policy Management, Johns Hopkins Bloomberg School of Public Health, Baltimore, MD, USA; n Division of Gastroenterology and Hepatology, Department of Internal Medicine, Toho University Sakura Medical Center, Sakura, Chiba, Japan

**Keywords:** Crohn's disease, Inflammatory bowel disease, Patient-reported outcomes, Quality of life, Cluster analysis

## Abstract

**Background:**

Clinical symptoms do not necessarily align with disease activity in ulcerative colitis (UC). It remains unclear whether patient-reported outcomes (PROs), particularly quality of life (QOL) measures, can help characterize clinically meaningful subphenotypes among Japanese patients with UC in remission. This study aimed to identify and characterize UC subphenotypes using clustering analysis.

**Methods:**

We used data from a multicenter, prospective UC patient registry in Japan, called “YOu and Ulcerative colitis: Registry and Social network (YOURS)” (December 2018–June 2022). We assessed laboratory values and PRO data, including clinical symptoms and QOL data (e.g., fatigue, anxiety, disease-specific QOL), of patients with UC in remission. Discovery (N = 365) and replication (N = 982) datasets were independently created and subjected to clustering analysis. Data were standardized for sex (a potential confounder for various measures).

**Findings:**

PRO data correlated poorly with traditional clinical laboratory parameters. Three reproducible clusters were detected in both datasets: one cluster was characterized by lower QOL and average laboratory profiles, while the other two clusters showed preserved QOL scores either with or without distinct laboratory profiles. The cluster with lower QOL and average laboratory profiles demonstrated increased relapse rates during a 3-year follow-up in each dataset compared with the respective other two clusters.

**Interpretation:**

PRO assessments offer unique, clinically relevant data, distinct from routine biomarkers and clinical scores. Stratifying patients with UC using combined QOL and clinical laboratory parameters may help identify patients at higher risk of relapse and warrants further evaluation for its potential role in clinical decision-making.

**Funding:**

This study was funded by Takeda Pharmaceutical Company Limited.

**Research in context**

**Evidence before this study**

Patient stratification is essential in inflammatory bowel disease (IBD) for predicting prognosis, and review of the literature showed that published work mainly relies on clinical data and biomarkers, such as cytokine panels, microbiome profiling, and autoantibodies. While deep learning and computational models have expanded retrospective identification capabilities, causality and real-world predictive power remain limited. Emerging research across various conditions suggests that integrating patient-reported outcomes (PROs), including quality of life (QOL) measures, provides valuable insight, as these may reflect underlying biological processes and gut–brain interactions. Therefore, PRO-based stratification may have potential for more precise patient classification in IBD beyond traditional biomarkers.

**Added value of this study**

Clustering analysis of clinical values, symptoms, and QOL data among Japanese patients with UC in remission identified three main patient subphenotypes: one type in which traditional laboratory profiles and QOL measures were average; another type in which traditional laboratory profiles were distinct, but QOL was average; and, finally, a type with average laboratory profiles but lower QOL. The third subphenotype was associated with more disease flare-ups than the other subphenotypes, suggestive of poor prognosis.

**Implications of all the available evidence**

Our findings underline the limitations of clinical metrics in predicting relapses in patients with UC and suggest that QOL assessments can offer unique, clinically relevant information distinct from routine clinical profiles. Based on this work and previous research, integration of PROs should become standard practice in the management of patients with UC in remission to help improve long-term outcomes.

## Introduction

Ulcerative colitis (UC) is a chronic inflammatory bowel disease (IBD) that affects the colon and rectum and is designated as an intractable disease in Japan.^1^^,^^2^ Although the incidence of UC is lower in Japan than in Western countries, its incidence is increasing in Japan, which may increase the burden on society.^3^ In Japan, the prevalence of IBD reached 254.8 patients per 100,000 people in 2023, while in North America and Europe, a prevalence of 156–291 patients per 100,000 people has been reported for the periods 1996–2002 and 1986–2000.^2^^,^^4^

The reasons for the increase in the incidence of UC remain unclear, although it has been shown to be associated with environmental factors, such as diet and lifestyle, as well as an aging population.^1^ There are three major categories of burdens related to UC. First, the physical burden of UC includes diarrhea, fatigue, stomach pain, rectal bleeding, and other colorectal discomforts.^5^ Second, due to the chronic nature of the disease, UC also heavily impacts mental health, with patients experiencing anxiety and depression related to their condition.^6^ Finally, the financial burden is taxing at both individual and societal levels.^7^ Therefore, as a disease associated with high physical, mental, and financial burdens, as well as increasing prevalence, improvements in the care and treatment of UC should be a priority.

One of the key challenges in IBD, including UC, is that clinical symptoms often do not correlate with underlying disease activity.^8^ Many patients report irritable bowel syndrome (IBS)-like symptoms or abdominal pain even during remission, complicating treatment strategies.^9^ Notably, psychological comorbidities are underdiagnosed and undertreated, despite evidence showing that they significantly increase the healthcare costs and are associated with poorer prognosis.^10^ On the other hand, patient-reported outcomes (PROs), such as quality of life (QOL) measures, have been shown to correlate with symptoms. Worsening of symptoms negatively impacts QOL, reflected as a negative impact on the patient's life and social activities.^11^

Data-driven clustering approaches have been used for various conditions to identify subphenotypes of the patient population and individuals with increased risks for complications.^12^^,^^13^ Recent research has begun to address the heterogeneity of the UC population through data-driven clustering approaches that incorporate both gastrointestinal symptoms and biomarkers, as well as psychological symptom profiles.^14^^,^^15^ Such model-based clustering methods, as previously applied in IBS, have shown promise in stratifying patients into clinically meaningful subgroups based on symptoms and mental health burdens.^16^ This stratification enables more precise predictions of disease outcomes and resource needs.

As clinical and demographic diversity among patients with UC is considerable, identifying subphenotypes based on factors related to physical, psychological, and lifestyle, as well as treatment history data, could lead to more personalized, efficient care.^17^^,^^18^ In particular, for patients in remission—who typically show no or only subtle changes in traditional biomarkers^19^—subphenotyping based on PROs may aid in predicting disease recurrence and guiding tailored interventions. Incorporating psychological assessment into standard UC management, alongside clustering-based approaches, represents a novel and impactful strategy to improve QOL, reduce societal burden, and advance personalized medicine in UC care. This study used a patient-centered registry to elucidate how lifestyle, psychosocial factors, and clinical practice patterns affect patient-centered outcomes.

## Methods

### Study design

This study was conducted using a multicenter, prospective patient registry conducted in Japan between December 2018 and June 2022, called “YOu and Ulcerative colitis: Registry and Social network (YOURS)” (UMIN Clinical Trials Registry: #UMIN000031995).^17^^,^^18^^,^^20^ YOURS is a patient-focused registry, recruiting 2006 consecutive patients with UC aged ≥16 years across five academic hospitals. This study represents the largest cohort study of PRO data from patients with UC in Japan.

Ethical approval was obtained from the ethics committees of the investigational sites: the Institute of Science Tokyo (IST), formerly called Tokyo Medical and Dental University, Medical Hospital (Tokyo, Japan; M2017-327-10); Kitasato University Kitasato Institute Hospital (KUKIH; Tokyo, Japan; 18010); Kyorin University Hospital (KUH; Tokyo, Japan; 1096); Tokyo Women's Medical University Hospital (TWMUH; Tokyo, Japan; 4817); and Toho University Medical Center Sakura Hospital (TUMC; Chiba, Japan; S18043). This study was conducted in accordance with the Declaration of Helsinki and all applicable Japanese laws and guidelines. Written informed consent was obtained from all patients prior to study enrollment.

### Study objectives

The objective of this study was to identify and characterize the subphenotypes within the UC patient population in Japan using clustering analysis of data from the YOURS database.

### Patient population

Patients with UC in remission, used as the only inclusion criterion, were selected from those enrolled in the YOURS study. Remission was defined as having a stool frequency score of 0 or 1 and a rectal bleeding score of 0 on the Two-Item Patient-Reported Outcomes (PRO-2). No exclusion criteria were applied.

The collected data were subdivided into discovery and replication datasets. The discovery dataset included data collected from IST, and the replication dataset comprised data from the other four institutions: KUH, TWMUH, TUMC, and KUKIH.

### Data collection

As part of the multicenter, prospective cohort study, patients with UC included in the YOURS dataset were annually surveyed using a paper questionnaire, and clinical and medical information was also collected annually from medical records for a total of 3 years. Further, information on stool frequency and rectal bleeding was collected using the Electronic Patient Reported Outcome (ePRO) application for smartphones every 3 months. If symptoms could not be registered using a smartphone, information was collected every 3 months via email, phone, or paper questionnaires. Details are provided in another YOURS-related article.

Baseline data used for the study included age, sex, body mass index (BMI), education, disease duration, history of medical treatments for UC, lifestyle factors such as sleep duration, social support, stress, total physical activity quantity, smoking history, years of smoking, disease-specific QOL, fatigue, anxiety, depression, pain, activity impairment, the amount of alcohol intake, medication adherence, complications, and pregnancy history.^17^ Laboratory measurements at baseline included albumin, hemoglobin (Hb), white blood cell (WBC), total cholesterol and C-reactive protein (CRP). The number of comorbidities was included as complications in the baseline data.

PRO questionnaires were used to obtain the baseline data: Pittsburgh Sleep Quality Index (PSQI) for sleep duration, modified Medical Outcomes Study Social Support Survey (mMOS-SS) for social support, Japanese version of the Perceived Stress Scale (JPSS) for stress, International Physical Activity Questionnaire (IPAQ) for total physical activity quantity in metabolic equivalents (METs) (minutes per week), Short Inflammatory Bowel Disease Questionnaire (SIBDQ) for disease-specific QOL, Functional Assessment of Chronic Illness Therapy-Fatigue (FACIT-F) for fatigue, Hospital Anxiety and Depression Scale (HADS) for anxiety and depression,^21^ Numerical Rating Scale (NRS) for pain, Work Productivity and Activity Impairment for activity impairment (WPAI-AI), Brief Self-Administered Diet History Questionnaire (BDHQ) for the amount of alcohol intake, and Adherence Starts with Knowledge 12 (ASK-12) for medication adherence.

Missing values in continuous variables such as clinical scores and laboratory measurements were imputed using the mean of the observed values. Categorical variables were excluded from the imputation process. In addition, where logical assumptions could be made based on demographic or contextual information, missing values were imputed accordingly to maintain internal consistency. For instance, male participants with missing pregnancy history were assumed to have no history of pregnancy, and participants under the age of 20 with missing smoking status were considered non-smokers. The variables in which data is missing in more than 30% patient records were excluded at the variable selection process.

### Study endpoints

The study endpoints were disease outcomes including flare-ups, hospitalization, and colectomy for each identified UC subphenotype. These three variables were assessed every 3 months during a 3-year follow-up period. Flare-ups were defined as follows: 1) if the stool frequency per day during the worst symptom episode (lasting 3 or more consecutive days) increased during the 90-day period compared with that before UC development, or 2) if rectal bleeding during the worst symptom episode (lasting 3 or more consecutive days) increased during the 90-day period from the last assessment. Hospitalization was counted if the patient had at least a new course of hospitalization since the previous assessment point. For colectomy, records for surgical procedures including “total (or subtotal) colectomy,” “ileal pouch-anal (analcanal) anastomosis,” and “creation of an artificial anus,” were counted only if the patient had at least one colectomy at the corresponding assessment point. For each of the three variables, statistical differences in trends were compared between clusters using the Kaplan–Meier analysis to measure the time to the first flare-up, colectomy, and hospitalization for each patient across the three clusters.

### Statistical analysis

Statistical analysis was conducted following three steps: “variable selection,” “cluster generation,” and “clustering analysis” In the “variable selection” process, baseline demographic, clinical, and PRO data from the YOURS database were inputted into an unsupervised clustering algorithm. Variables were initially selected by a clinical expert for relevance and to remove any overlapping or conflicting items. Subphenotypes of UC in the Japanese population were identified through a combination of Hierarchical Clustering on Principal Components (HCPC) and t-distributed stochastic neighbor embedding (t-SNE). A feature correlation matrix heatmap was developed using the continuous variables from the YOURS database as a reference for the clustering inputs. t-SNE was performed separately to visualize high-dimensional data in a more digestible format. One-hot encoding was used to convert categorical data into binary features for t-SNE. Factor Analysis of Mixed Data (FAMD) was used to identify the most important variables for subsequent cluster analysis. FAMD was then used to reduce the number of variables into fewer dimensions, and these dimensions were used for hierarchical clustering. The initial partition for the hierarchical clustering was then further partitioned through t-SNE.

The process of “cluster generation” is briefly summarized in Supplementary Fig. S1. First, standardization by sex was applied by subtracting their means and then dividing by their standard deviations within each sex group prior to the clustering process. Next, dimensionality reduction via FAMD was performed on the selected variables, yielding a set of uncorrelated factors that summarized the key information contained in the original variables. These factors were then used as the input for the subsequent cluster generation. Hierarchical clustering was applied to the reduced dimensions obtained from FAMD using Ward's criterion. The optimal number of clusters was determined by analyzing the hierarchical tree and evaluating inertia gain in the initial partition. Finally, the resulting clusters were visualized using t-SNE to facilitate interpretation and provide an intuitive representation of patient grouping. Both the original data and imputed data were utilized to generate clusters.

In the “clustering analysis” step, a descriptive statistics table was produced for all the studied measures included in the “cluster” generation step. Key factors representative for each cluster were visualized using radar charts, and the characteristics of each cluster were described using boxplots. Outcomes such as flare-ups, as well as hospitalization and colectomy, were compared between clusters using the Kaplan–Meier curve analysis during the follow-up period. Statistical differences between clusters were assessed using the log-rank test.

### Data management and quality assurance

Data cleaning and quality control were previously performed on the complete dataset. Data management was performed in accordance with standard operating procedures developed by the Data Management Division of the Contract Research Organization (CRO), which was commissioned by Takeda Pharmaceutical Company Limited, a joint research organization.

### Role of the funding source

Takeda Pharmaceutical Company Limited funded this study, assisted with designing the study, and supported data collection, analysis, and interpretation.

## Results

### Study population characteristics

Out of 2006 patients enrolled in the YOURS registry,^18^ 1347 patients with UC in remission at the time of enrollment were included in this study. Table 1 shows the patient demographics and baseline characteristics of the discovery (N = 365) and replication (N = 982) datasets. The median age was 42.0 years (interquartile range [IQR]: 32.0–52.0) in the discovery dataset and 45.0 years (IQR: 34.0–56.0) in the replication dataset. The percentage of female patients was 50.4% in the discovery dataset and 45.9% in the replication dataset. The median BMI was similar between the two datasets, with a median of 21.4 kg/m^2^ (IQR: 19.9–23.8) in the discovery dataset and 21.8 kg/m^2^ (IQR: 19.9–24.1) in the replication dataset.

**Table 1:** Patient demographics and baseline characteristics of the discovery (N = 365) and replication (N = 982) datasets.

	Discovery dataset (N = 365)	Replication dataset (N = 982)
Demographics		
Age (years)		
Median (IQR)	42.0 (32.0–52.0)	45.0 (34.0–56.0)
Sex		
Male	181 (49.6%)	531 (54.1%)
Female	184 (50.4%)	451 (45.9%)
BMI (kg/m^2^)		
Median (IQR)	21.4 (19.9–23.8)	21.8 (19.9–24.1)
Education		
Elementary or junior high school	2 (0.5%)	20 (2.0%)
High school	37 (10.1%)	180 (18.3%)
Vocational school	42 (11.5%)	130 (13.2%)
Junior college	28 (7.7%)	89 (9.1%)
University	128 (35.1%)	408 (41.5%)
Postgraduate	24 (6.6%)	58 (5.9%)
Disease characteristics		
Disease duration (years)		
Median (IQR)	8.4 (5.3–13.8)	10.6 (5.5–16.5)
History of treatment		
5-ASA	320 (87.7%)	935 (95.2%)
Tacrolimus	6 (1.6%)	40 (4.1%)
Cyclosporine	0 (0.0%)	14 (1.4%)
Infliximab	52 (14.2%)	103 (10.5%)
Adalimumab	22 (6.0%)	71 (7.2%)
Golimumab	3 (0.8%)	26 (2.6%)
Vedolizumab	0 (0.0%)	4 (0.4%)
Leukocytapheresis	8 (2.2%)	81 (8.2%)
Lifestyle		
Sleep duration (hours)		
Median (IQR)	6.0 (6.0–7.0)	6.5 (6.0–7.0)
Social support (mMOS-SS)		
Median (IQR)	75.0 (56.3–87.5)	75.0 (56.3–87.5)
Stress (JPSS)		
Median (IQR)	24.0 (20.0–29.0)	23.0 (19.0–28.0)
Total MET (minutes/week)		
Median (IQR)	1188.0 (495.0–2772.0)	1412.5 (594.0–2970.0)
Have you ever smoked?		
Yes	133 (36.4%)	388 (39.5%)
No	232 (63.6%)	594 (60.5%)
Years of smoking		
Median (IQR)	12.0 (6.0–21.0)	15.0 (10.0–25.0)
SIBDQ score		
Median (IQR)	5.8 (5.2–6.2)	5.9 (5.4–6.4)
FACIT_F score		
Median (IQR)	43.0 (38.0–47.0)	44.0 (39.0–48.0)
HADS_ANX score		
Median (IQR)	4.0 (2.0–7.0)	4.0 (2.0–6.0)
HADS_DP score		
Median (IQR)	3.0 (1.0–6.0)	3.0 (1.0–5.0)
NRS score		
Median (IQR)	0.0 (0.0–1.0)	0.0 (0.0–1.0)
WPAI_AI score		
Median (IQR)	0.0 (0.0–10.0)	0.0 (0.0–10.0)
PSQI score		
Median (IQR)	5.0 (4.0–7.0)	5.0 (3.0–7.0)
Alcohol (g/day)		
Median (IQR)	48.2 (0.0–108.0)	55.5 (0.0–108.0)
Laboratory data		
Albumin (g/dL)		
Median (IQR)	4.3 (4.2–4.4)	4.3 (4.2–4.4)
Hemoglobin (g/dL)		
Median (IQR)	13.9 (12.9–14.5)	13.9 (13.4–14.5)
White blood cell count (10^3^/μL)		
Median (IQR)	5.6 (4.6–5.9)	5.6 (4.9–5.9)
C-reactive protein (mg/dL)		
Median (IQR)	0.1 (0.0–0.2)	0.1 (0.0–0.2)

5-ASA: 5-aminosalicylic acid; BMI: body mass index; FACIT_F: Functional Assessment of Chronic Illness Therapy-Fatigue; HADS_ANX: Hospital Anxiety and Depression Scale-Anxiety; HADS_DP: Hospital Anxiety and Depression Scale-Depression; IQR: interquartile range; JPSS: Japanese version of the Perceived Stress Scale; MET: metabolic equivalent; mMOS-SS: modified Medical Outcomes Study Social Support Survey; NRS: Numerical Rating Scale; PSQI: Pittsburgh Sleep Quality Index; SD: standard deviation; SIBDQ: Short Inflammatory Bowel Disease Questionnaire; WPAI_AI: Work Productivity and Activity Impairment-Activity Impairment.

The median disease duration at the time of inclusion in the study was 8.4 years (IQR: 5.3–13.8) in the discovery dataset and 10.6 years (IQR: 5.5–16.5) in the replication dataset. The patients had a history of treatment with 5-aminosalicylic acid (5-ASA) in 87.7% and 95.2%, with steroids in 26.6% and 38.2%, infliximab in 14.2% and 10.5%, in the discovery and replication set, respectively. Limited numbers of patients had a history of treatment with tacrolimus, cyclosporine, adalimumab, golimumab, vedolizumab, or leukocytapheresis therapy (range: 0.0%–8.2%).

### Clinical data clustering

**Fig. 1:**
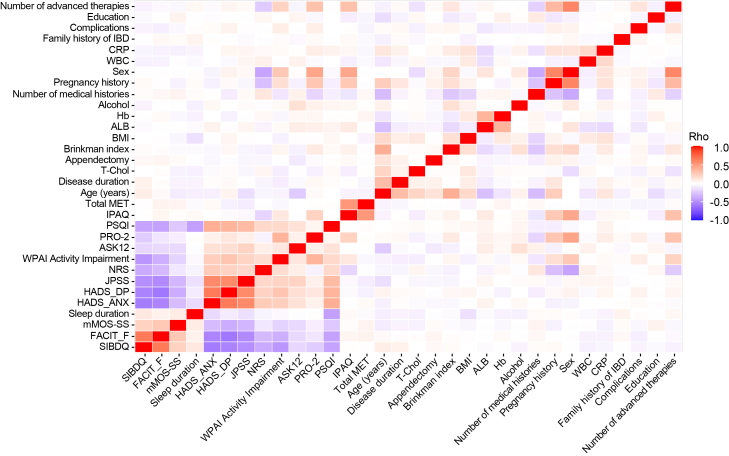
**Correlation matrix heatmap of data clustering of clinical and PRO measures**. ALB: albumin; ASK-12: Adherence Starts with Knowledge 12; BMI: body mass index; CRP: C-reactive protein; FACIT_F: Functional Assessment of Chronic Illness Therapy-Fatigue; HADS_ANX: Hospital Anxiety and Depression Scale-Anxiety; HADS_DP: Hospital Anxiety and Depression Scale-Depression; Hb: hemoglobin; IBD: inflammatory bowel disease; IPAQ: International Physical Activity Questionnaire; JPSS: Japanese version of the Perceived Stress Scale; MET: metabolic equivalent; mMOS-SS: modified Medical Outcomes Study Social Support Survey; NRS: Numerical Rating Scale; PRO: patient-reported outcome; PRO-2: Two-Item Patient-Reported Outcomes; PSQI: Pittsburgh Sleep Quality Index; SIBDQ: Short Inflammatory Bowel Disease Questionnaire; T-Chol: total cholesterol; WBC: white blood cell; WPAI: Work Productivity and Activity Impairment.

The heatmap in Fig. 1 represents the clustering of clinical data and summarizes the correlation among various data variables, including blood test results and PRO data. In the heatmap, an area with high correlation can be observed on the bottom left, representing PRO data. These PRO data correlated poorly with traditional clinical values, such as WBC counts and CRP levels. This suggests that patients with stable WBC counts and low CRP levels, typically characteristic of stable disease, may still experience impairment in PRO measures.

### Clustering analysis

**Fig. 2:**
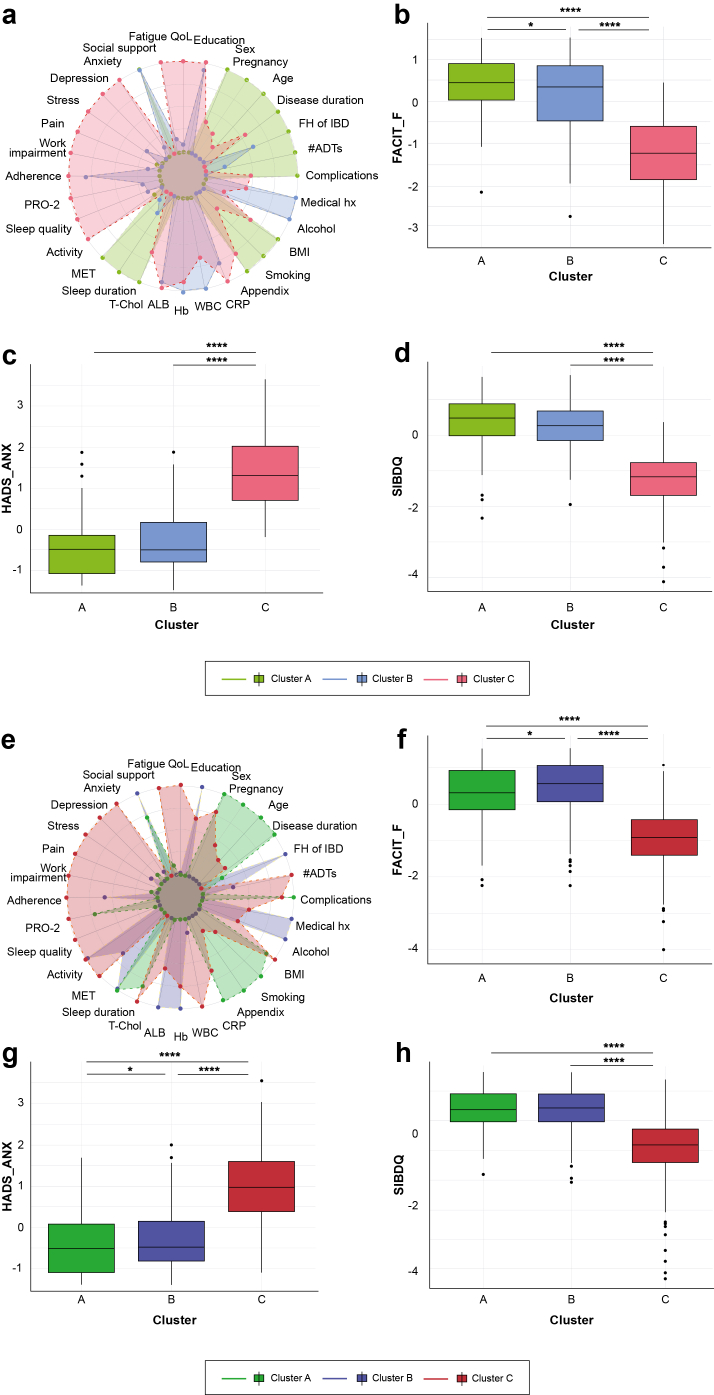
**Clustering analysis**. a) Radar plot for the discovery dataset, b) FACIT_F scores by cluster in the discovery dataset, c) HADS_ANX scores by cluster in the discovery dataset, d) SIBDQ scores by cluster in the discovery dataset, e) Radar plot for the replication dataset, f) FACIT_F scores by cluster in the replication dataset, g) HADS_ANX scores by cluster in the replication dataset, h) SIBDQ scores by cluster in the replication dataset. Lower FACIT_F and SIBDQ (QOL) scores and higher HADS_ANX, NRS (pain), WPAI-AI, and JPSS (stress) scores indicate worse health states. See Supplementary Fig. 2 for additional visualizations. Radar charts and boxplots of the clusters derived from the hierarchical clustering analysis are shown for each dataset. For the radar plots, the mean value of each variable for each cluster was calculated, and the plot was drawn based on these cluster-specific mean values. Values closer to the center represent the minimum mean value of any cluster, while values farther from the center represent the maximum mean value. For SIBDQ and FACIT_F scores, with lower scores indicating lower QOL or greater fatigue, the lower scores were placed farther from the center, and the higher values were placed closer to the center. Both continuous variables, age and laboratory values, and categorical variables, sex (male: 1, female: 2) and presence/absence (absent: 0, present: 1), were averaged. (∗p < 0.05, ∗∗∗∗p < 0.001; Tukey's multiple comparison test). #ADT: number of advanced therapies used for ulcerative colitis; ALB: albumin; BMI: body mass index; CRP: C-reactive protein; FACIT_F: Functional Assessment of Chronic Illness Therapy-Fatigue; FH of IBD: family history of inflammatory bowel disease; HADS_ANX: Hospital Anxiety and Depression Scale-Anxiety; Hb: hemoglobin; hx: history; MET: metabolic equivalent; PRO-2: Two-Item Patient-Reported Outcomes; QOL: quality of life; SIBDQ: Short Inflammatory Bowel Disease Questionnaire; T-Chol: total cholesterol; WBC: white blood cell.

Supplementary Fig. S2 shows the t-SNE visualization for both datasets, revealing three main clusters. In Fig. 2 and Supplementary Fig. S3, radar charts and boxplots of the derived clusters based on the t-SNE data are shown for each dataset. The variables used for clustering are shown in Fig. 2.

Three clusters were detected in each dataset, based on their respective characteristics regarding laboratory profiles and PRO parameters. Patients in Cluster B (blue) had generally average laboratory profiles and QOL, while those in Cluster A (green) had distinct laboratory profiles but average QOL. Finally, patients in Cluster C (red) had average laboratory profiles but worse QOL in terms of anxiety, depression, stress, pain, activity impairment, sleep quality, and social support, compared with the other two clusters in their respective dataset (Fig. 2). Supplementary Table S1 lists the differences in characteristics among the three clusters. Similar data were found between the discovery and replication datasets. Patterns were comparable between the datasets, and cluster profiles remained distinguishable across multiple patient-reported and objective measures, supporting robustness of the clustering approach. When comparing the most prominent factors, after standardizing for sex, age was identified as the most frequent contributing factor to the cluster formation (Supplementary Fig. S4), with higher age observed in Cluster A (green) (Supplementary Table S1).

### Clinical outcomes

**Fig. 3:**
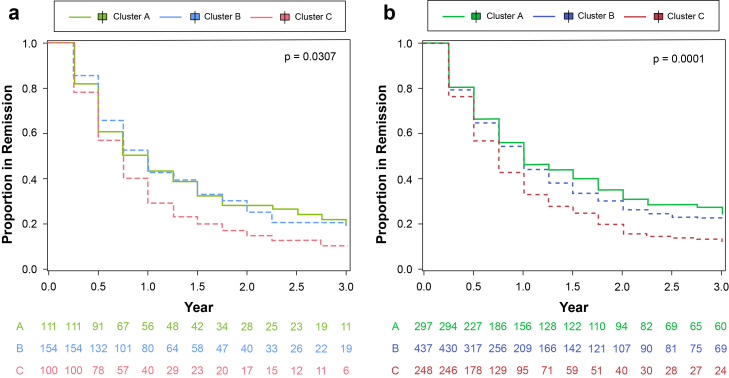
**Time to first flare-up over the study period for each cluster**. a) Discovery dataset, b) Replication dataset. Time to first flare-up: The number at risk includes individuals who experienced the event on that day.

For further assessing outcomes among the clusters, we performed an analysis of flare-ups of UC symptoms, hospitalizations, and colectomy. Fig. 3 shows Kaplan–Meier curves representing the time to the first flare-up since study entry for all the included patients within a cluster. In the discovery dataset, 63/111, 80/111, and 86/111 patients of Cluster A had their first flare-ups in Years 1, 2, and 3, respectively. Moreover, 88/154, 113/154, and 120/154 patients of Cluster B and 71/100, 85/100, and 89/100 patients of Cluster C had their first flare-ups in Years 1, 2, and 3, respectively. Similarly, in the replication dataset, 236/437, 305/437, and 319/437 patients of Cluster B had the first flare-ups, while 159/297, 202/297, and 216/297 patients of Cluster A and 163/248, 201/248, and 208/248 patients of Cluster C had the first flare-up in each year. Overall event rates were relatively high across all phenotypes, likely reflecting the sensitive PRO-based definition of flare. As shown in Fig. 3, the difference in median survival was statistically significant among the clusters in the discovery dataset (p = 0.0307) and those in the replication dataset (p = 0.0001). Thus, patients in Cluster C had a shorter time to the first flare-up than those in the other two clusters in their respective dataset. Therefore, these data suggest that the patients with UC in remission from the clusters with worse QOL but average laboratory profiles had a higher likelihood of relapse. As for colectomy, in the discovery dataset, a total of 1/111, 1/154 and 0/100 patient each in Cluster A, Cluster B, and Cluster C, respectively, underwent colectomy for 3 years. Notably, no patient in the analysis cohort of either dataset had undergone a total colectomy. Likewise, in the replication dataset, a total of 3/437 patients in Cluster B, 4/297 in Cluster A, and 0/248 in Cluster C underwent colectomy during the 3-year follow-up period. There were no significant differences among Cluster A, Cluster B, and Cluster C in the discovery dataset or among Cluster B, Cluster A, and Cluster C in the replication dataset (p = 0.6606 for discovery dataset; p = 0.1767 for replication dataset). With regard to hospitalization, in the discovery dataset, 5/111 patients in Cluster A, 6/154 in Cluster B, and 5/100 in Cluster C experienced hospitalization. Similarly, in the replication dataset, 17/437 patients in Cluster B, 20/297 in Cluster A, and 10/248 in Cluster C experienced hospitalization for 3 years. The differences in median survival among the clusters in the discovery and those in the replication datasets were not significant (p = 0.9120 for discovery dataset; 0.1694 for replication dataset). The lack of significant differences in hospitalization and colectomy among the three clusters of each dataset may be attributed to insufficient event counts for each outcome, limiting the statistical power to detect such differences as well as the possibility that PRO-defined clusters primarily capture symptomatic rather than structural disease burden.

## Discussion

In the previous YOURS database study, PROs were shown to be important factors for patients with UC, with PROs impacted by symptom severity.^18^ Even patients with mild UC experienced impaired QOL and reduced work productivity. In the current study, we performed clustering analysis to identify subphenotypes among Japanese patients with UC in remission to help stratify patients for appropriate care and to predict outcomes. Our analyses identified three main clusters based on inertia gain in both datasets, of which one cluster was associated with average laboratory profiles and average PROs, another cluster was associated with distinct laboratory profiles but average PROs, and the third cluster had average laboratory profiles but worse PROs. The third cluster was associated with more flare-ups than the other clusters, suggesting poor prognosis, even though these patients were in remission and exhibited stable clinical metrics at study enrollment. These findings underline the limitations of routine clinical metrics in UC for predicting relapse.

Previous studies have used a variety of PRO outcomes, including clinical and QOL outcome measures, to stratify patients with IBD into subgroups and identify patients at an increased risk for flare-ups, requiring more care, and with worse mental health outcomes.^14^^,^^22^^,^^23^ We detected low correlations between several traditional systemic biomarkers, such as CRP levels and WBC counts, and the scores obtained by PRO for QOL. Therefore, routine clinical measures may not fully reflect ongoing disease burden in clinically stable patients.

To identify subpopulations with favorable or poor outcomes, stratification is essential and has previously been performed for IBD and other diseases.^13^^,^^14^^,^^24^ For these types of analyses in IBD, previous work included clinical demographics and biomarkers, such as multiplex cytokine panels, microbiome, autoantibodies, and conventional blood tests, to predict disease development and treatment response.^25^^,^^26^ While recent advances in deep learning have allowed for retrospective analyses of clinical trial data to stratify patients with poor prognosis, the prospective utility of such approaches remains limited.^27^ PROs may capture subjective well-being and serve as an indicator of underlying biological processes, currently not captured by these methods.

We validated our data by assessing two cohorts, a discovery dataset and a replication dataset. Analyses were performed separately for each dataset, and similar clustering data were obtained, confirming the data. In order to improve care for patients with UC in remission, the inclusion of PROs, such as QOL and clinical symptoms, in treatment planning and monitoring will be useful. This was also outlined in STRIDE II, which suggested inclusion of PROs as a treatment target on top of clinical outcome measures.^28^ Therefore, closer monitoring of QOL may help improve care and aid in preventing relapses. Further, identification of clusters with worse QOL could enable selecting patients for targeted psychological or supportive interventions aimed at reducing future relapses.

We detected higher fatigue, anxiety, stress, activity impairment, pain, and lower social support in one of the clusters. This highlights the possible need for multidisciplinary care approaches including mental health support for these patients. Previous studies have also highlighted worse QOL and higher psychological and stress scores among patients with UC.^14^^,^^18^ Recent data from the YOURS study showed that among patients with UC in remission, many patients adopt lifestyle restrictions associated with impaired QOL but not with reduced relapses.^20^ Van der Eijk et al. showed a clear relationship between QOL in patients with IBD, including UC, and the quality of healthcare they received, particularly related to the information provided by, costs of, and courtesy from healthcare providers.^29^ Although impaired QOL was associated with an increased risk of relapse, whether improving QOL directly reduces relapse risk remains uncertain. While chronic stress has been linked to disease flare and psychological interventions can improve QOL, no robust evidence currently demonstrates that QOL improvement alone alters disease course. QOL should therefore be interpreted primarily as a prognostic marker. Improving care, particularly for patients in worse QOL clusters, may therefore be a targeted approach to improve outcomes, and prospective interventional studies are warranted.

Beyond the prognostic implications, the reproducibility of the clustering structure across two independent datasets suggests that symptom-based phenotyping may capture persistent differences in disease experience among patients with UC in remission. Patients with impaired QOL formed a distinct cluster despite having normal biomarkers, indicating that PRO-derived clusters reflect dimensions that extend beyond biochemical or endoscopic metrics. Although speculative, these PRO-based subphenotypes may represent the aggregate, or “net effect,” of subtle, multifactorial disease activity. Further investigation into these pathways may identify modifiable contributors to relapse. Integrating symptom- and QOL-based stratification alongside endoscopic and biomarker assessments may support a more comprehensive definition of remission, narrowing the gap between biological control and lived disease burden.

PRO-derived subphenotypes highlight clinically meaningful variations not captured by the current biomarker frameworks, underscoring the need for biomarkers that better reflect patient experience. Existing measures, including CRP, WBC count, and even fecal calprotectin, may not fully detect subtle or multidimensional disease activity associated with impaired well-being in remission. As remission was defined using PRO-2 criteria, residual inflammatory activity cannot be excluded; however, this may also reflect real-world clinical settings and suggests that PRO-based stratification can identify patients with potential subclinical disease activity despite apparent remission. Furthermore, while the prospective design supports QOL as a prognostic marker, reverse causality cannot be excluded, as prodromal or subclinical disease activity may contribute to impaired QOL. Although advances in molecular profiling, neuroimmune signaling markers, microbiome-based signatures, and digital health-derived physiologic metrics have been explored, their clinical utility remains limited and insufficiently validated.^25^, ^26^, ^27^ Identifying novel biomarkers that correlate with QOL-defined phenotypes could support earlier detection of deterioration, guide personalized treatment strategies, and refine the definition of deep remission to encompass both biological control and patient experience. Future studies integrating multi-omics platforms with structured longitudinal PRO datasets will be essential to determine markers that accurately track these symptom-based disease states.

Future prospective studies should further validate the detected patient subphenotypes and evaluate targeted interventions aimed specifically at improving PROs. Additionally, implementation of simplified, yet comprehensive, QOL assessment tools could enhance routine clinical decision-making and patient care for UC.

While we used prospective multicenter patient registry data, included a large sample size, specifically collected PRO data of Japanese patients with UC, and validated data in separate datasets, our study has some limitations. First, clinical remission was defined solely using PRO-2 criteria to avoid selection bias arising from limited availability of endoscopic data at baseline and to ensure broader applicability to real-world clinical settings. Therefore, the possibility of residual subclinical inflammation cannot be excluded. Second, although clustering structures were reproducible across independent cohorts, due to patient recruitment from specialized, high-volume medical centers, there is potential for selection bias of patients receiving a level of care that might not be available to all patients with UC across other continents and racial groups. Similar discovery-validation frameworks have been widely adopted in data-driven subphenotyping studies, in which subgroups identified in a single cohort were subsequently validated in independent external cohorts.^30^ Third, mean imputation was applied for missing continuous variables, which may reduce variance and influence correlation structure, particularly in clustering analyses. In addition, variable selection was based on clinical judgment, and sex standardization was applied to adjust for known differences; however, these approaches may introduce bias or reduce biological variability. Furthermore, the analytical framework involved several data-driven decisions including variable and clustering parameter selection; therefore, these methodological choices may have influenced cluster formation and the interpretation of relapse risk. Fourth, outcome analyses were based on unadjusted comparisons, and potential confounding factors such as age, disease duration, and treatment history were not accounted for. Therefore, the observed associations between cluster membership and relapse risk should be interpreted as descriptive rather than indicative of independent predictive effects. Fifth, hospitalization events were not restricted to UC exacerbations due to the registry design, and the relatively low number of hospitalization and colectomy events may have limited statistical power to detect differences across clusters. Furthermore, age contributed substantially to cluster formation, and the extent to which clusters reflect age-related comorbidity or psychosocial factors rather than disease-specific subphenotypes cannot be fully determined. Finally, longitudinal analyses could provide deeper insights into the temporal dynamics and generalizability of the data. Future studies incorporating serial assessments, including endoscopic evaluation and biomarker measurements such as fecal calprotectin, can be expected to determine whether cluster membership is stable over time or shifts dynamically in response to treatment, lifestyle influences, or fluctuations in inflammatory or symptomatic burden.

### Conclusions

Our study shows that QOL assessments offer unique, clinically relevant information that is distinct from routine biomarkers and clinical scores. Stratifying patients with UC using combined PROs and clinical parameter data may help identify those at increased risk or relapse. Integrating PROs, and in particular QOL data, into routine clinical practice may enhance patient-centered management in patients with UC in remission and warrants further evaluation for its potential role in improving long-term outcomes.

## Contributors

YM, HY, and KM contributed to the conception and design of the study. TH, MN, TK, MY, TO, and KM contributed to the acquisition of data. YM directly accessed and verified the underlying data reported in the manuscript. YM accessed and verified data. All authors contributed to data analysis and interpretation, as well as revision of the manuscript for important intellectual content. All authors had full access to all the data in the study, accepted responsibility for submission, and gave final approval for the manuscript to be published.

## Data sharing statement

De-identified individual participant data that underlie the results reported in this article (including text, tables, figures, and appendices) will be shared, along with the study protocol. Data will be shared to investigators whose proposed use of individual participant data for meta-analysis has been approved by an independent review committee (“learned intermediary”) identified for this purpose. Data will be available for sharing from 9 months to 36 months following article publication. After 36 months the data will be available upon request to jimukyoku@jsibd.jp, but without investigator support other than deposited metadata information regarding submitting proposals.

## Declaration of interests

All authors received support for the present manuscript from Takeda Pharmaceutical. HY received lecture fees from Janssen Pharmaceutical, Mitsubishi Tanabe Pharma, Kowa, AstraZeneca, Kyorin Pharmaceutical, and Takeda Pharmaceutical. Under contracts with Kyoto University, fees for consultation with HY were paid to Kyoto University by Takeda Pharmaceutical and Magmitt Pharmaceutical. TH received grants from Mitsubishi Tanabe Pharma, EA Pharma, AbbVie, JIMRO, Zeria Pharmaceutical, Kyorin Pharmaceutical, Nippon Kayaku, Takeda Pharmaceutical, Pfizer, Boston Scientific, and Mochida Pharmaceutical; consulting fees from EA Pharma, AbbVie, Janssen Pharmaceutical, Pfizer, Mitsubishi Tanabe Pharma, JIMRO, Mochida Pharmaceutical, Bristol-Myers Squibb, Eli Lilly, Gilead Sciences, and Abivax; and lecture fees from EA Pharma, AbbVie, Janssen Pharmaceutical, Pfizer, Mitsubishi Tanabe Pharma, JIMRO, Mochida Pharmaceutical, Bristol-Myers Squibb, Eli Lilly, and Gilead Sciences. TK received research grants from AbbVie, Alfresa, EA Pharma, Gilead Sciences, Nippon Kayaku, Eli Lilly, Mochida Pharmaceutical, Janssen Pharmaceutical, Pfizer, Sekisui Medical, Samsung Medical, Takeda Pharmaceutical, Bristol-Myers Squibb, Mitsubishi Tanabe Pharma, Zeria Pharmaceutical, JIMRO, and Helmsley Charitable Trust; consulting fees from Takeda Pharmaceutical, Alfresa, Zeria Pharmaceutical, Kyorin Pharmaceutical, Nippon Kayaku, Mitsubishi Tanabe Pharma, AbbVie, Pfizer, Janssen Pharmaceutical, JIMRO, and Galapagos; and honoraria from AbbVie, EA Pharma, Mitsubishi Tanabe Pharma, Takeda Pharmaceutical, Pfizer, Janssen Pharmaceutical, Mochida Pharmaceutical, Kissei Pharmaceutical, Nippon Kayaku, and Bristol-Myers Squibb. SS received grants from AbbVie, EA Pharma, JIMRO, Kyorin Pharmaceutical, Nippon Kayaku, Mochida Pharmaceutical, and Zeria Pharmaceutical; consulting fees from AbbVie, Brystol-Myers Squibb, Celltrion Healthcare, EA Pharma, Eli Lilly, Gilead Sciences, Janssen Pharmaceutical, Kissei Pharmaceutical, Sekisui Medical, and Takeda Pharmaceutical; and honoraria from AbbVie, Celltrion Healthcare, EA Pharma, Gilead Sciences, Janssen Pharmaceutical, JIMRO, Kissei Pharmaceutical, Kyorin Pharmaceutical, Mitsubishi Tanabe Pharma, Mochida Pharmaceutical, Nippon Kayaku, Pfizer, Sekisui Medical, and Takeda Pharmaceutical. TF received grants from AbbVie, Alfresa, Boehringer Ingelheim, Bristol-Myers Squibb, Celgene, Celltrion Healthcare, EA Pharma, Eli Lilly, Gilead Sciences, Janssen Pharmaceutical, Kissei Pharmaceutical, Mebix, Sanofi, and Takeda Pharmaceutical; received honoraria from AbbVie, Boehringer Ingelheim, Bristol-Myers Squibb, Daiichi Sankyo, EA Pharma, Janssen Pharmaceutical, Kissei Pharmaceutical, Kyowa Hakko Kirin, Mitsubishi Tanabe Pharma, Mochida Pharmaceutical, Nichi-iko, Nippon Kayaku, Pfizer, Takeda Pharmaceutical, Taiho Pharmaceutical, and Zeria Pharmaceutical; and serves as Councilor for the Japanese Society of Gastroenterology. MS received grants from Mochida Pharmaceutical, Janssen Pharmaceutical, Bristol Myers Squibb, MSD, and Celltrion Healthcare Japan, and honoraria from Janssen Pharmaceutical, AbbVie, EA Pharma, Bristol Myers Squibb, Takeda Pharmaceutical, Mochida Pharmaceutical, and Gilead Sciences. SM received grants from Janssen Pharmaceutical and honoraria from AbbVie, Mochida Pharmaceutical, Gilead Sciences, and Janssen Pharmaceutical. TY received honoraria from AbbVie and Pfizer. MM received honoraria from Janssen Pharmaceutical, AbbVie, Takeda Pharmaceutical, Pfizer, Mochida Pharmaceutical, Kyorin Pharmaceutical, Bristol-Myers Squibb, EA Pharma, Mitsubishi Tanabe Pharma, Celltrion Healthcare, Nippon Kayaku, JIMRO, and Kissei Pharmaceutical. TO received honoraria from Takeda Pharmaceutical and AbbVie. KM received grants from Mochida Pharmaceutical, AbbVie, and EA Pharma and honoraria from Takeda Pharmaceutical, Mitsubishi Tanabe Pharma, Janssen Pharmaceutical, AbbVie, EA Pharma, Pfizer, Mochida Pharmaceutical, Kissei Pharmaceutical, Gilead Sciences, Bristol-Myers Squibb, and Eli Lilly. YM, MN, MY, and SF declare no conflicts of interest.
