# Supplementary material for: Data-driven subphenotyping uncovers ulcerative colitis subtype with high risk for relapse in Japan: a prospective multicenter cohort study
Source: Lancet Reg Health West Pac. 2026 Jul 24;73:101932. doi: 10.1016/j.lanwpc.2026.101932 (PMC13427516; doi:10.1016/j.lanwpc.2026.101932)
Supplement: Supplementary Figures and Tables. [file mmc1.docx]

**SUPPLEMENTARY MATERIAL**

Table of Contents

[Supplementary Fig. S1: Cluster generation process 2](#_Toc216173122)

[Supplementary Fig. S2: Clustering analysis. 3](#_Toc216173123)

[Supplementary Fig. S3: Scores of PRO variables in each cluster. 4](#_Toc216173124)

[Supplementary Fig. S4: Contribution of variables in clustering. 5](#_Toc216173125)

[Supplementary Table S1: Characteristics of each cluster in the two datasets (Standardized Data) 7](#_Toc216173126)


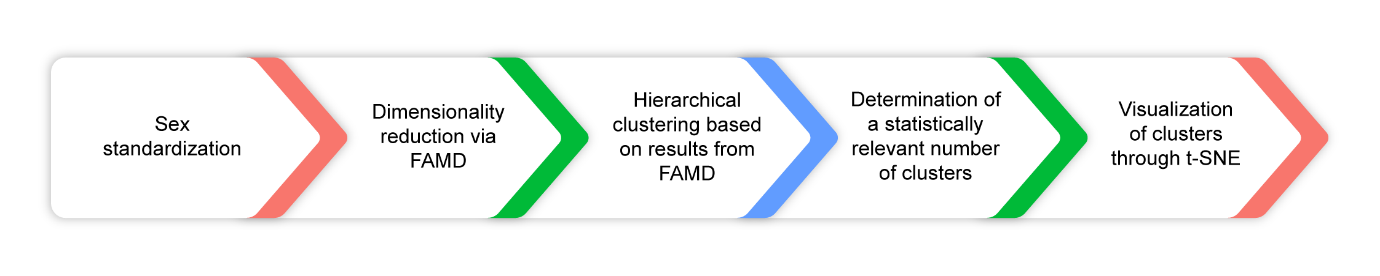


# Supplementary Fig. S1: Cluster generation process

*FAMD:* *Factor Analysis of Mixed Data; t-SNE: t-distributed stochastic neighbor embedding*

Supplementary Fig. S2: Clustering analysis. a) t-SNE visualization of the discovery dataset, b) representation of correlation between features and principal dimensions in the discovery dataset, c) t-SNE visualization of the replication dataset, and d) representation of correlation between features and principal dimensions in the replication dataset.

*ALB, albumin; ASK-12: Adherence Starts with Knowledge 12; BMI: body mass index; CRP: C-reactive protein; FACIT_F: Functional Assessment of Chronic Illness Therapy-Fatigue; HADS_ANX: Hospital Anxiety and Depression Scale-Anxiety; HADS_DP: Hospital Anxiety and Depression Scale-Depression^1^; Hb, hemoglobin; ^2^: Japanese version of the Perceived Stress Scale; LB, laboratory; MET: metabolic equivalent; mMOS-SS: modified Medical Outcomes Study Social Support Survey; NRS: Numerical Rating Scale; PSQI: Pittsburgh Sleep Quality Index^3-5^; SIBDQ: Short Inflammatory Bowel Disease Questionnaire^6^; T-Chol, total cholesterol; t-SNE: t-distributed stochastic neighbor embedding; WPAI: Work Productivity and Activity Impairment^7^*

**

Supplementary Fig. S3: Scores of PRO variables in each cluster. a) JPSS scores by cluster in the discovery dataset, b) WPAI scores by cluster in the discovery dataset, c) JPSS scores by cluster in the replication dataset, and d) WPAI scores by cluster in the replication dataset. (*p<0.05, ****p<0.001; Tukey’s multiple comparison test)

Higher JPSS (stress) and WPAI (Activity Impairment) scores indicate worse health states.

*JPSS: Japanese version of the Perceived Stress Scale; WPAI: Work Productivity and Activity Impairment–Activity Impairment*


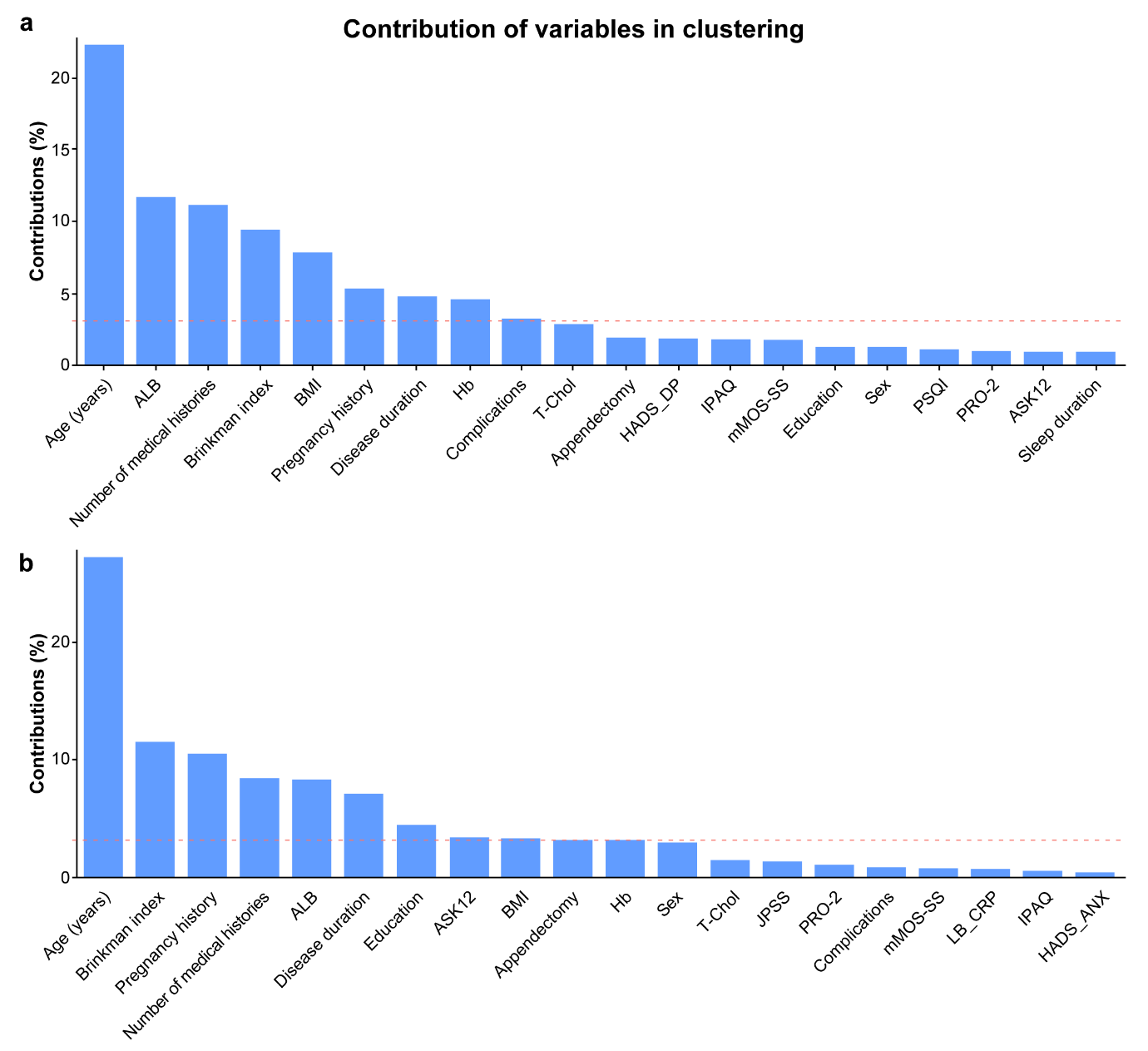


Supplementary Fig. S4: Contribution of variables in clustering. a) Discovery dataset, b) Replication dataset.

*ALB: albumin; ASK-12: Adherence Starts with Knowledge 12; BMI: body mass index; CRP: C-reactive protein; HADS_ANX: Hospital Anxiety and Depression Scale-Anxiety; HADS_DP: Hospital Anxiety and Depression Scale-depression; Hb: hemoglobin; IPAQ: International Physical Activity Questionnaire^8,9^; JPSS: Japanese version of the Perceived Stress Scale; LB: lab variable; ^10^-SS: modified Medical Outcomes Study Social Support Survey; PRO-2: Two-Item Patient-Reported Outcomes; T-Chol: total cholesterol*

*Number of medical histories includes any previous or current comorbidity, including hypertension, angina and myocardial infarction, heart failure, diabetes mellitus, stroke, asthma, chronic obstructive pulmonary disease, chronic kidney disease, rheumatoid arthritis/collage disease, osteoarthritis, osteoporosis, gastric/duodenal ulcer, chronic hepatitis/cirrhosis, or depression.*

# Supplementary references:

1. Zigmond AS, Snaith RP. The Hospital Anxiety and Depression Scale. *Acta Psychiatrica Scandinavica* 1983; **67**(6): 361-70.

2. Mimura C, Griffiths P. A Japanese version of the Perceived Stress Scale: cross-cultural translation and equivalence assessment. *BMC Psychiatry* 2008; **8**: 85.

3. Buysse DJ, Reynolds CF, III, Monk TH, Berman SR, Kupfer DJ. The Pittsburgh Sleep Quality Index: a new instrument for psychiatric practice and research. *Psychiatry Research* 1989; **28**(2): 193-213.

4. Doi Y, Minowa M, Okawa M, Uchiyama M. Development of the Japanese version of the Pittsburgh Sleep Quality Index. *Japanese Journal of Psychiatry Treatment* 1998; **13**(6): 755-63.

5. Doi Y, Minowa M, Uchiyama M, et al. Psychometric assessment of subjective sleep quality using the Japanese version of the Pittsburgh Sleep Quality Index (PSQI-J) in psychiatric disordered and control subjects. *Psychiatry Research* 2000; **97**(2-3): 165-72.

6. Irvine EJ, Zhou Q, Thompson AK. The Short Inflammatory Bowel Disease Questionnaire: a quality of life instrument for community physicians managing inflammatory bowel disease. CCRPT Investigators. Canadian Crohn's Relapse Prevention Trial. *The American Journal of Gastroenterology* 1996; **91**(8): 1571-8.

7. Reilly MC, Zbrozek AS, Dukes EM. The validity and reproducibility of a work productivity and activity impairment instrument. *PharmacoEconomics* 1993; **4**(5): 353-65.

8. Craig CL, Marshall AL, Sjöström M, et al. International Physical Activity Questionnaire: 12-Country Reliability and Validity. *Medicine & Science in Sports & Exercise* 2003; **35**(8): 1381-95.

9. Murase N, Katsumura T, Ueda C, Inoue S, Shimomitsu T. Validity and reliability of Japanese version of International Physical Activity Questionnaire. *Journal of Health and Welfare Statistics* 2002; **49**(11): 1-9.

10. Togari T, Yokoyama Y. Application of the eight-item modified medical outcomes study social support survey in Japan: a national representative cross-sectional study. *Quality of Life Research* 2016; **25**(5): 1151-8.

**Supplementary Table S1**

| Supplementary Table S1: Characteristics of each cluster in the two datasets (Standardized Data) | | | | | | | | | | | | | | |
| --- | --- | --- | --- | --- | --- | --- | --- | --- | --- | --- | --- | --- | --- | --- |
|  | **Discovery Dataset** | | | | | | | **Replication Dataset** | | | | | | |
|  | **Cluster 1**  **(N=111)** | **Cluster 2**  **(N=154)** | **Cluster 3**  **(N=100)** | **Cluster 1 vs· 2**  **p-value** | **Cluster 1 vs· 3**  **p-value** | **Cluster 2 vs· 3**  **p-value** | **Overall p-value** | **Cluster I**  **(N=437)** | **Cluster II2**  **(N=297)** | **Cluster III**  **(N=248)** | **Cluster 1 vs· 2**  **p-value** | **Cluster 1 vs· 3**  **p-value** | **Cluster 2 vs· 3**  **p-value** | **Overall p-value** |
| **Demographics** |  |  |  |  |  |  |  |  |  |  |  |  |  |  |
|  |  |  |  |  |  |  |  |  |  |  |  |  |  |  |
| **Age (years)** |  |  |  |  |  |  |  |  |  |  |  |  |  |  |
| Mean (SD) | 0·6  (0·89) | -0·5  (0·72) | -0·3  (0·86) | <0·0001 | <0·0001 | 0·1101 | <0·0001 | -0·5 (0·73) | 0·9 (0·79) | 0·0 (0·91) | <0·0001 | <0·0001 | <0·0001 | <0·0001 |
|  |  |  |  |  |  |  |  |  |  |  |  |  |  |  |
| **Sex** |  |  |  |  |  |  |  |  |  |  |  |  |  |  |
| Male | 26 (23·4%) | 104 (67·5%) | 51 (51·0%) | <0·0001 | <0·0001 | 0·0083 | <0·0001 | 278 (63·6%) | 132 (44·4%) | 121 (48·8%) | <0·0001 | 0·0002 | 0·3110 | <0·0001 |
| Female | 85 (76·6%) | 50 (32·5%) | 49 (49·0%) |  |  |  |  | 159 (36·4%) | 165 (55·6%) | 127 (51·2%) |  |  |  |  |
|  |  |  |  |  |  |  |  |  |  |  |  |  |  |  |
| **BMI (kg/m^2^)** |  |  |  |  |  |  |  |  |  |  |  |  |  |  |
| Mean (SD) | 0·1  (0·94) | -0·1 (0·99) | 0·0 (1·26) | 0·1473 | 0·7934 | 0·5010 | 0·1608 | -0·2 (0·85) | 0·2 (1·01) | 0·2 (1·08) | <0·0001 | <0·0001 | 0·8641 | <0·0001 |
|  |  |  |  |  |  |  |  |  |  |  |  |  |  |  |
| **Education** |  |  |  |  |  |  |  |  |  |  |  |  |  |  |
| Elementary or junior high school | 0 (0·0%) | 0 (0·0%) | 2 (2·0%) | <0·0001 | 0·0135 | 0·1484 | 0·0002 | 7 (1·6%) | 6 (2·0%) | 7 (2·8%) | <0·0001 | 0·0004 | <0·0001 | <0·0001 |
| High school | 19 (17·1%) | 9  (5·8%) | 9  (9·0%) |  |  |  |  | 45 (10·3%) | 88 (29·6%) | 47 (19·0%) |  |  |  |  |
| Vocational school | 10  (9·0%) | 20 (13·0%) | 12 (12·0%) |  |  |  |  | 61 (14·0%) | 23  (7·7%) | 46 (18·5%) |  |  |  |  |
| Junior college | 15 (13·5%) | 5  (3·2%) | 8  (8·0%) |  |  |  |  | 29  (6·6%) | 36 (12·1%) | 24  (9·7%) |  |  |  |  |
| University | 25 (22·5%) | 61 (39·6%) | 42 (42·0%) |  |  |  |  | 222 (50·8%) | 91 (30·6%) | 95 (38·3%) |  |  |  |  |
| Postgraduate | 4  (3·6%) | 15  (9·7%) | 5  (5·0%) |  |  |  |  | 39  (8·9%) | 9  (3·0%) | 10  (4·0%) |  |  |  |  |
| Missing | 38 (34·2%) | 44 (28·6%) | 22 (22·0%) |  |  |  |  | 34  (7·8%) | 44 (14·8%) | 19  (7·7%) |  |  |  |  |
|  |  |  |  |  |  |  |  |  |  |  |  |  |  |  |
| **Disease characteristics** |  |  |  |  |  |  |  |  |  |  |  |  |  |  |
|  |  |  |  |  |  |  |  |  |  |  |  |  |  |  |
| **Disease duration (years)** |  |  |  |  |  |  |  |  |  |  |  |  |  |  |
| Mean (SD) | 0·1  (1·00) | -0·3   (0·72) | -0·1  (0·81) | 0·0010 | 0·2894 | 0·1442 | 0·0015 | -0·3 (0·77) | 0·6 (1·27) | 0·0 (0·95) | <0·0001 | 0·0009 | <0·0001 | <0·0001 |
|  |  |  |  |  |  |  |  |  |  |  |  |  |  |  |
| **History of treatment** |  |  |  |  |  |  |  |  |  |  |  |  |  |  |
| 5-ASA^#^ | 52 (46·8%) | 71 (46·1%) | 52 (52·0%) | 0·9283 | 0·4938 | 0·4112 | 0·6879 | 278 (63·6%) | 183 (61·6%) | 161 (64·9%) | 0·9532 | 0·5254 | 0·5250 | 0·7768 |
| 5-ASA^#^ | 44 (39·6%) | 63 (40·9%) | 39 (39·0%) | 0·8140 | 0·8823 | 0·6986 | 0·9243 | 214 (49·0%) | 121 (40·7%) | 119 (48·0%) | 0·0605 | 0·9635 | 0·1102 | 0·1334 |
| 5-ASA^#^ | 30 (27·0%) | 42 (27·3%) | 39 (39·0%) | 0·9488 | 0·0708 | 0·0587 | 0·1043 | 109 (24·9%) | 60 (20·2%) | 70 (28·2%) | 0·1939 | 0·2842 | 0·0337 | 0·1046 |
| Steroids | 31 (27·9%) | 30 (19·5%) | 36 (36·0%) | 0·1104 | 0·2248 | 0·0041 | 0·0159 | 158 (36·2%) | 111 (37·4%) | 106 (42·7%) | 0·5276 | 0·0594 | 0·2393 | 0·1682 |
| Tacrolimus | 1 (0·9%) | 2 (1·3%) | 3 (3·0%) | 0·7601 | 0·2683 | 0·3483 | 0·4510 | 23 (5·3%) | 9 (3·0%) | 8 (3·2%) | 0·1683 | 0·2337 | 0·9158 | 0·2708 |
| Cyclosporine | 0 (0·0%) | 0 (0·0%) | 0 (0·0%) |  |  |  |  | 8 (1·8%) | 4 (1·3%) | 2 (0·8%) | 0·6456 | 0·2931 | 0·5376 | 0·5673 |
| Infliximab | 16 (14·4%) | 24 (15·6%) | 12 (12·0%) | 0·7823 | 0·5879 | 0·4003 | 0·7018 | 47 (10·8%) | 22  (7·4%) | 34 (13·7%) | 0·1588 | 0·2179 | 0·0178 | 0·0614 |
| Adalimumab | 8 (7·2%) | 7 (4·5%) | 7 (7·0%) | 0·3590 | 0·9389 | 0·4168 | 0·6061 | 30 (6·9%) | 20 (6·7%) | 21 (8·5%) | 0·9732 | 0·4066 | 0·4683 | 0·6713 |
| Golimumab | 1 (0·9%) | 1 (0·6%) | 1 (1·0%) | 0·8177 | 0·9460 | 0·7646 | 0·9520 | 13 (3·0%) | 6 (2·0%) | 7 (2·8%) | 0·4593 | 0·9380 | 0·5555 | 0·7486 |
| 5-ASA^#^ | 5 (4·5%) | 8 (5·2%) | 9 (9·0%) | 0·7860 | 0·1885 | 0·2407 | 0·3340 | 58 (13·3%) | 70 (23·6%) | 32 (12·9%) | 0·0002 | 0·9459 | 0·0011 | 0·0001 |
| Vedolizumab | 0 (0·0%) | 0 (0·0%) | 0 (0·0%) |  |  |  |  | 0 (0·0%) | 1 (0·3%) | 3 (1·2%) | 0·2187 | 0·0203 | 0·2394 | 0·0551 |
| Leukocytapheresis | 2 (1·8%) | 3 (1·9%) | 3 (3·0%) | 0·9277 | 0·5748 | 0·6011 | 0·8162 | 37 (8·5%) | 20 (6·7%) | 24 (9·7%) | 0·4509 | 0·5472 | 0·2239 | 0·4777 |
|  |  |  |  |  |  |  |  |  |  |  |  |  |  |  |
| **Lifestyle** |  |  |  |  |  |  |  |  |  |  |  |  |  |  |
|  |  |  |  |  |  |  |  |  |  |  |  |  |  |  |
| **Sleep duration (hours)** |  |  |  |  |  |  |  |  |  |  |  |  |  |  |
| Mean (SD) | 0·2 (0·99) | -0·1 (0·88) | -0·2 (1·20) | 0·0385 | 0·0105 | 0·7342 | 0·0084 | 0·1 (0·94) | 0·1 (0·96) | -0·4 (0·99) | 0·9851 | <0·0001 | <0·0001 | <0·0001 |
|  |  |  |  |  |  |  |  |  |  |  |  |  |  |  |
| **Social support (mMOS-SS)** |  |  |  |  |  |  |  |  |  |  |  |  |  |  |
| Mean (SD) | 0·2  (0·85) | 0·2 (0·93) | -0·6 (1·03) | 0·9855 | <0·0001 | <0·0001 | <0·0001 | 0·3 (0·80) | 0·1 (1·03) | -0·6 (1·02) | 0·0005 | <0·0001 | <0·0001 | <0·0001 |
|  |  |  |  |  |  |  |  |  |  |  |  |  |  |  |
| **Stress (JPSS)** |  |  |  |  |  |  |  |  |  |  |  |  |  |  |
| Mean (SD) | -0·5  (0·80) | -0·1 (0·79) | 1·2 (0·89) | 0·0017 | <0·0001 | <0·0001 | <0·0001 | -0·3 (0·76) | -0·5 (0·80) | 0·9 (0·84) | 0·0050 | <0·0001 | <0·0001 | <0·0001 |
|  |  |  |  |  |  |  |  |  |  |  |  |  |  |  |
| **Total MET (minutes/week)** |  |  |  |  |  |  |  |  |  |  |  |  |  |  |
| Mean (SD) | 0·0 (1·07) | -0·1 (0·87) | -0·1 (0·88) | 0·5895 | 0·5728 | 0·9907 | 0·5235 | 0·0 (0·95) | 0·0 (1·00) | 0·1 (1·17) | 0·7525 | 0·9193 | 0·5837 | 0·5959 |
|  |  |  |  |  |  |  |  |  |  |  |  |  |  |  |
| **Have you ever smoked?** |  |  |  |  |  |  |  |  |  |  |  |  |  |  |
| Yes | 39 (35·1%) | 61 (39·6%) | 33 (33·0%) | 0·4584 | 0·7439 | 0·2864 | 0·5324 | 141 (32·3%) | 157 (52·9%) | 90 (36·3%) | <0·0001 | 0·2678 | 0·0001 | <0·0001 |
| No | 72 (64·9%) | 93 (60·4%) | 67 (67·0%) |  |  |  |  | 296 (67·7%) | 140 (47·1%) | 157 (63·3%) |  |  |  |  |
|  |  |  |  |  |  |  |  |  |  |  |  |  |  |  |
| **Years of smoking** |  |  |  |  |  |  |  |  |  |  |  |  |  |  |
| Mean (SD) | 1·6 (1·23) | 0·3 (0·84) | 0·8 (1·10) | <0·0001 | 0·0070 | 0·0641 | <0·0001 | 0·4 (0·68) | 1·4 (1·25) | 0·8 (1·10) | <0·0001 | 0·0170 | <0·0001 | <0·0001 |
|  |  |  |  |  |  |  |  |  |  |  |  |  |  |  |
| **SIBDQ** |  |  |  |  |  |  |  |  |  |  |  |  |  |  |
| Mean (SD) | 0·4 (0·74) | 0·2 (0·66) | -1·3 (0·82) | 0·2904 | <0·0001 | <0·0001 | <0·0001 | 0·4 (0·68) | 0·4 (0·69) | -1·0 (1·05) | 0·7803 | <0·0001 | <0·0001 | <0·0001 |
|  |  |  |  |  |  |  |  |  |  |  |  |  |  |  |
| **FACIT_F** |  |  |  |  |  |  |  |  |  |  |  |  |  |  |
| Mean (SD) | 0·4  (0·67) | 0·2 (0·83) | -1·3 (0·87) | 0·0395 | <0·0001 | <0·0001 | <0·0001 | 0·5 (0·71) | 0·3 (0·76) | -1·0 (0·87) | 0·0150 | <0·0001 | <0·0001 | <0·0001 |
|  |  |  |  |  |  |  |  |  |  |  |  |  |  |  |
| **HADS_ANX** |  |  |  |  |  |  |  |  |  |  |  |  |  |  |
| Mean (SD) | -0·5  (0·68) | -0·3  (0·75) | 1·3 (0·83) | 0·0692 | <0·0001 | <0·0001 | <0·0001 | -0·3 (0·72) | -0·4 (0·68) | 1·0 (0·91) | 0·2036 | <0·0001 | <0·0001 | <0·0001 |
|  |  |  |  |  |  |  |  |  |  |  |  |  |  |  |
| **HADS_DP** |  |  |  |  |  |  |  |  |  |  |  |  |  |  |
| Mean (SD) | -0·4 (0·64) | -0·4  (0·65) | 1·3 (0·88) | 0·8653 | <0·0001 | <0·0001 | <0·0001 | -0·4 (0·63) | -0·3 (0·76) | 1·0 (0·98) | 0·0345 | <0·0001 | <0·0001 | <0·0001 |
|  |  |  |  |  |  |  |  |  |  |  |  |  |  |  |
| **NRS** |  |  |  |  |  |  |  |  |  |  |  |  |  |  |
| Mean (SD) | -0·2 (0·67) | -0·2 (0·61) | 0·8 (1·49) | 0·8850 | <0·0001 | <0·0001 | <0·0001 | -0·2 (0·62) | -0·2 (0·82) | 0·7 (1·31) | 1·0000 | <0·0001 | <0·0001 | <0·0001 |
|  |  |  |  |  |  |  |  |  |  |  |  |  |  |  |
| **WPAI_AI** |  |  |  |  |  |  |  |  |  |  |  |  |  |  |
| Mean (SD) | -0·3 (0·45) | -0·1 (0·79) | 0·9 (1·56) | 0·4020 | <0·0001 | <0·0001 | <0·0001 | -0·3 (0·49) | -0·2 (0·65) | 0·7 (1·42) | 0·4715 | <0·0001 | <0·0001 | <0·0001 |
|  |  |  |  |  |  |  |  |  |  |  |  |  |  |  |
| **PSQI** |  |  |  |  |  |  |  |  |  |  |  |  |  |  |
| Mean (SD) | -0·4 (0·71) | -0·2 (0·73) | 0·8 (1·12) | 0·1765 | <0·0001 | <0·0001 | <0·0001 | -0·3 (0·79) | -0·3 (0·83) | 0·8 (1·09) | 0·7512 | <0·0001 | <0·0001 | <0·0001 |
|  |  |  |  |  |  |  |  |  |  |  |  |  |  |  |
| **Alcohol (g/day)** |  |  |  |  |  |  |  |  |  |  |  |  |  |  |
| Mean (SD) | -0·2 (0·69) | 0·1 (1·12) | -0·1 (0·71) | 0·0157 | 0·8427 | 0·0916 | 0·0126 | 0·1 (1·19) | -0·1 (0·80) | 0·0 (0·97) | 0·1936 | 0·6223 | 0·7889 | 0·2145 |
|  |  |  |  |  |  |  |  |  |  |  |  |  |  |  |
| **Laboratory data** |  |  |  |  |  |  |  |  |  |  |  |  |  |  |
|  |  |  |  |  |  |  |  |  |  |  |  |  |  |  |
| **Albumin (g/dL)** |  |  |  |  |  |  |  |  |  |  |  |  |  |  |
| Mean (SD) | -0·4 (0·93) | 0·2 (0·86) | 0·3 (1·05) | <0·0001 | <0·0001 | 0·9139 | <0·0001 | 0·3 (0·83) | -0·5 (1·05) | 0·0 (1·04) | <0·0001 | <0·0001 | <0·0001 | <0·0001 |
|  |  |  |  |  |  |  |  |  |  |  |  |  |  |  |
| **Hemoglobin (g/dL)** |  |  |  |  |  |  |  |  |  |  |  |  |  |  |
| Mean (SD) | -0·3 (0·71) | 0·2 (0·81) | 0·1 (1·87) | 0·0024 | 0·0168 | 0·9502 | 0·0020 | 0·2 (0·98) | -0·3 (0·84) | 0·0 (0·80 | <0·0001 | 0·2002 | <0·0001 | <0·0001 |
|  |  |  |  |  |  |  |  |  |  |  |  |  |  |  |
| **White blood cell (10^3^/μL)** |  |  |  |  |  |  |  |  |  |  |  |  |  |  |
| Mean (SD) | -0·4 (0·81) | 0·1 (0·87) | -0·1 (0·83) | 0·0003 | 0·0558 | 0·3801 | 0·0005 | 0·0 (0·98) | 0·0 (1·02) | 0·1 (1·17) | 0·9500 | 0·2884 | 0·2217 | 0·2045 |
|  |  |  |  |  |  |  |  |  |  |  |  |  |  |  |
| **C-reactive protein (mg/dL)** |  |  |  |  |  |  |  |  |  |  |  |  |  |  |
| Mean (SD) | -0·1 (0·47) | -0·1 (0·63) | -0·1 (0·48) | 0·8955 | 0·8591 | 0·9907 | 0·8536 | -0·1 (0·44) | 0·2 (1·61) | 0·1 (1·22) | 0·0008 | 0·0659 | 0·4935 | 0·0009 |
| *5-ASA: 5-aminosalicylic acid; BMI: body mass index; FACIT_F: Functional Assessment of Chronic Illness Therapy - Fatigue; HADS_ANX: Anxiety of Hospital Anxiety and Depression Scale; HADS_DP: Hospital Anxiety and Depression Scale-Depression; JPSS: Japanese version of the Perceived Stress Scale; MET: metabolic equivalent; mMOS-SS: modified Medical Outcomes Study Social Support Survey; NRS: Numerical Rating Scale; PSQI: Pittsburgh Sleep Quality Index; SD: standard deviation; SIBDQ: Short Inflammatory Bowel Disease Questionnaire; WPAI_AI: Work Productivity and Activity Impairment-Activity Impairment*  *#Combined figures of four 5-ASA treatments were not available.* | | | | | | | | | | | | | | |
